# Decellularized Dental Pulp, Extracellular Vesicles, and 5-Azacytidine: A New Tool for Endodontic Regeneration

**DOI:** 10.3390/biomedicines10020403

**Published:** 2022-02-08

**Authors:** Francesca Diomede, Luigia Fonticoli, Guya Diletta Marconi, Ylenia Della Rocca, Thangavelu Soundara Rajan, Oriana Trubiani, Giovanna Murmura, Jacopo Pizzicannella

**Affiliations:** 1 Department of Innovative Technologies in Medicine & Dentistry, University “G. d’Annunzio” Chieti-Pescara, Via dei Vestini, 31, 66100 Chieti, Italy; francesca.diomede@unich.it (F.D.); luigia.fonticoli@unich.it (L.F.); ylenia.dellarocca@unich.it (Y.D.R.); 2 Department of Medical, Oral and Biotechnological Sciences, University “G. d’Annunzio” Chieti-Pescara, Via dei Vestini, 31, 66100 Chieti, Italy; guya.marconi@unich.it; 3 Department of Biotechnology, Karpagam Academy of Higher Education, Coimbatore 641021, India; drsoundararajan.t@kahedu.edu.in; 4 Ss. Annunziata” Hospital, ASL 02 Lanciano-Vasto-Chieti, 66100 Chieti, Italy; jacopo.pizzicannella@unich.it

**Keywords:** decellularized dental pulp, extracellular vesicles, 5-azacytidine, endodontic regeneration, extracellular matrix scaffold

## Abstract

Dental pulp is a major component of the dental body that serves to maintain the tooth life and function. The aim of the present work was to develop a system that functions as a growth-permissive microenvironment for dental pulp regeneration using a decellularized dental pulp (DDP) matrix, 5-Aza-2′-deoxycytidine (5-Aza), and Extracellular Vesicles (EVs) derived from human Dental Pulp Stem Cells (hDPSCs). Human dental pulps extracted from healthy teeth, scheduled to be removed for orthodontic purpose, were decellularized and then recellularized with hDPSCs. The hDPSCs were seeded on DDP and maintained under different culture conditions: basal medium (CTRL), EVs, 5-Aza, and EVs+-5-Aza. Immunofluorescence staining and Western blot analyses were performed to evaluate the proteins’ expression related to dentinogenesis, such as ALP, RUNX2, COL1A1, Vinculin, DMP1, and DSPP. Protein contents found in the DDP recellularized with hDPSCs were highly expressed in samples co-treated with EVs and 5-Aza compared to other culture conditions. This study developed a DDP matrix loaded by hDPSCs in co-treatment with EVs, which might enhance the dentinogenic differentiation with a high potentiality for endodontic regeneration.

## 1. Introduction

Teeth life and homeostasis are maintained by dental pulp, which can be damaged by caries, pulpitis, injury, and pulp necrosis. Often when dental pulp is affected, pulpal necrosis is an obvious consequence [1,2]. To ameliorate the quality of life, dental pulp damage must be restored in order to maintain the functionality of the pulp-dentin complex.

When dental pulp is affected by trauma or caries, the most used treatment is root canal therapy, which leads to the loss of pulp tissue functions. After this type of treatment, teeth no longer survive. Tissue engineering could represent a valid alternative strategy to ‘save’ the teeth and enable dental pulp regeneration.

Several biomaterials have been used to provide a three-dimensional (3D) scaffold platform to promote tissue regeneration, including polylactic acid, collagen, hyaluronic acid, and chitosan [3,4]. However, to date there is no viable scaffold available that reproduces the dental pulp microenviroment by inducing the direct differentiation of stem cells.

In recent literature, it has been reported that decellularized extracellular matrices (dECMs) could be the gold standard scaffold material for tissue regeneration in heart, liver, nerves, and tendons [5,6]. In dentistry, the use of dECMs, in particular those derived from dental pulp tissue, was described for the stimulation of odontoblastic differentiation without exogenous growth or differentiation factors [7].

5-Aza-2′-deoxycytidine (5-Aza) acts as a reductor of DNA methylation through the inhibition of DNA methyltransferases (DNMTs) activity [8]. DNMT enzymes play a key role in cell proliferation and differentiation by regulating epigenetic modifications. 5-Aza is able to induce the odontogenic differentiation of hDPSCs without the use of odontogenic medium, as previously demonstrated [9]. The treatment with 5-Aza improved hDPSCs odontogenic differentiation, inhibited cell proliferation, and enhanced the expression of odontogenic related markers, such as DSPP and DMP1, RUNX2, DLX5, OSX, and ALP activity.

The odontogenic-related genes showed a reduction of DNA methylation that upregulated their expression in differentiated hDPSCs. The role of DNA demethylation during the odontogenic differentiation process of hDPCs may open new strategies for dental pulp repair, regeneration, and therapy [10].

In recent years, Extracellular Vesicles (EVs) have been represented as a new biosignaling paradigm. They are divided into two different categories: exosomes and microvesicles. They are responsible for cell communication via cargo transport of carrying the signaling proteins, RNA species, DNA, and lipids. Once released, EVs act as paracrine factors, as their content is selectively taken by near or distant target cells, which influences their behavior [11,12]. They also represent a safe alternative to stem cell therapy with the administration of whole cells. Stem-cell-based therapies have been shown to obtain promising results in tissue regeneration [13,14]; however, stem-cell transplantation might be associated with some clinical complications and ethical concerns [15].

EVs isolated from oral-cavity-derived Mesenchymal Stem Cells (MSCs) have been demonstrated to vehicle pro-angiogenic and anti-inflammatory factors [16]. The role of EVs has been studied in the enhancement of bone tissue regeneration via the induction of MSC differentiation towards osteogenic precursor cells. The exploration of the possibility to use the EVs isolated from hDPSCs as biomimetic tools to lead the odontogenic differentiation lineage is necessary to better comprehend the role of lineage for specific EVs to initiate the differentiation process.

In the present study adn for the first time to our knowledge, we developed a system constituted by human decellularized dental pulp (DDP) and used it as an ECM 3D scaffold enriched with hDPSCs, EVs, and 5-Aza that lead to a progressive cellular differentiation in cells with an odontoblastic phenotype in order to promote dental pulp tissue regeneration.

## 2. Materials and Methods

### 2.1. Cell Culture Establishment

The primary human DPSCs used in this study were isolated in the Dental Clinics of the Medical School of University “G. d’Annunzio” Chieti-Pescara. Human DPSCs were collected from a third molar scheduled to be extracted for orthodontic treatment as previously reported [17]. Tissue fragments were cultured for one week in a humidified and controlled atmosphere of 37 °C and 5% CO_2_ with MSCGM-CD medium (mesenchymal stem-cell growth medium chemically defined) (Lonza, Basel, Switzerland). Cells spontaneously migrated from tissue fragments were subcultured until P2. The medium was refreshed every two days. The cells used in all the experiments were at passage 2. All experiments were performed in triplicate.

### 2.2. EVs Isolation

Human DPSCs were seeded at confluence in 150 mm cell culture dishes. EVs were isolated from the culture medium of cells cultured for 48 h. EVs were isolated as per the previously published protocol [18]. Briefly, the EVs from the supernatant (starting volume 10 mL) were isolated using the ExoQuick-TC (System Biosciences, Euroclone SpA, Milan, Italy) following the manufacturer’s protocol. 2 mL of ExoQuick TC solution were added to 10 mL of conditioned medium, recovered as mentioned earlier. The mix was incubated overnight at 4 °C without rotation; one centrifugation step was performed at 1500× *g* for 30 min to sediment the EVs and the pellets were resuspended in 200 μL PBS. The detection of EV whole homogenate protein was used as a confirmation for the release of EVs from hDPSCs.

### 2.3. Samples Preparation

#### 2.3.1. Decellularization

Dental pulp tissues were obtained from extracted third molars for orthodontic purpose. Dental pulps (DP) were washed with a cleaning solution comprising Dulbecco’s phosphate-buffered saline (DPBS, Lonza, Basel, Switzerland) and antibiotics (1% penicillin/streptomycin, Lonza). To decellularize DP to obtain a decellularized dental pulp (DDP), 1% sodium dodecyl sulfate and 1% Triton X–100 (Sigma-Aldrich, Milan, Italy) in DPBS was used [19]. The solution was changed every 9 h during the procedure (total time procedure 48 h) and during the procedure tissues were kept in a continuous flutter. A post-decellularization cleaning procedure (DPBS + 1% penicillin/streptomycin) was performed for the following 48 h (solution were changed every 9 h).

To evaluate the morphological difference between DP and DDP by light microscopy, tissues were fixed in 10% (*w*/*v*) PBS-buffered formaldehyde followed by embedded in paraffin and sectioned into 7-mm thin slices. After processing into xylene deparaffinization and subsequent rehydration steps, sections were stained with toluidine blue and visualized under a light microscope (Leica Microsystem, Milan, Italy) connected to a high-resolution digital camera DFC425B Leica (Leica Microsystem).

To evaluate the morphological difference between DP and DDP by confocal microscopy, samples were fixed in 4% paraformaldehyde (PFA) (BioOptica, Milan, Italy) for 1 h at RT. Then, samples were permeabilized with 1% Triton X-100 (BioOptica) for 5 min and treated with blocking buffer made of 2% BSA in PBS for 1 h. Later, samples were incubated with TOPRO (1:200; ThermoFisher) for 1 h at 37 °C. Samples were observed with Zeiss LSM800 META confocal system (Zeiss, Jena, Germany) connected to an inverted Zeiss Axiovert 200 microscope equipped with a Plan Neofluar oil-immersion objectives.

#### 2.3.2. Recellularization

DDPs were recellularized for 15 days by hDPSCs and maintained in incubator at 37 °C and 5% CO_2_ in air; cells were seeded at the concentration of 1 × 10^6^ cells/mL. The culture medium was changed every 48 h. At the end of the recellularization period, morphological analyses were performed by microscopy analyses.

### 2.4. Study Design

The present research study was organized in the following experimental groups:

− Human DPSCs (CTRL)
− Human DPSCs treated with 5-Aza (5-Aza)
− Human DPSCs treated with EVs (EVs)
− Human DPSCs treated with EVs+5-Aza (5-Aza+EVs)
− DDP + Human DPSCs (DDP-CTRL)
− DDP + Human DPSCs treated with 5-Aza (DDP-5-Aza)
− DDP + Human DPSCs treated with EVs (DDP-EVs)
− DDP + Human DPSCs treated with EVs+5-Aza (DDP-5-Aza+EVs)

Treatment with 5-Azacytidine (5-Aza): Human DPSCS and hDPSCs cultured on DDP were treated with 5-Aza (5 mM) for 14 days in a humidified atmosphere 5% CO_2_ at 37 °C. Treatment with Extracellular Vescicles (EVs): Human DPSCS and hDPSCs cultured on DDP were treated with collected EVs for 14 days in a humidified atmosphere 5% CO_2_ at 37 °C. Treatment with EVs and 5-Aza: Human DPSCS and hDPSCs cultured on DDP were treated with EVs and 5-Aza for 14 days in a humidified atmosphere 5% CO_2_ at 37 °C.

5-Aza was purchased from Sigma-Aldrich (Merck, Milan, Italy) and was used at a concentration of 1 μmol/L. EVs were used at a final concentration of 24 μg.

### 2.5. Haematoxylin-Eosin and Himmunoistochemical Staining

All samples were fixed in 10% phosphate-buffered formalin for 2.5 h. Sections were de-waxed (Bioclear and alcohol in progressively lower concentrations), rehydrated, and processed for haematoxylin-eosin staining [20].

Paraffin-embedded tissue slices were deparaffinized with xylene, rehydrated with alcohol series, and incubated in 0.01 M citrate buffer (pH 6) for 4 min to retrieve antigen. Slices were then incubated with 0.3% (*v*/*v*) hydrogen peroxide in 60% (*v*/*v*) methanol for 30 min to quench endogenous peroxidase and were blocked with normal goat serum in PBS [2% (*v*/*v*)] for 20 min. Next, slices were incubated with selective primary antibodies overnight at 4 °C. The primary antibody applied for immunohistochemical analysis anti- DSPP (1:100 in PBS (*v*/*v*); SantaCruz Biotechnology, Santa Cruz, CA, USA)) was used. Slices were then washed with PBS and incubated with avidin/biotin blocking reagent (DBA, Milan, Italy) to block endogenous avidin and biotin binding sites. Next, slices were incubated with universal biotinylated secondary Ab followed by avidin horseradish peroxidase (HRP)–conjugated solution (Vectastain ABC Kit; Vector Laboratories, Burlingame, CA, USA) according to manufacturer’s instructions. Slices were then incubated with a hydrogen peroxide/DAB kit (Vector Laboratories, DBA, Milan, Italy) according to the manufacturer’s instructions. Counterstaining was performed with haematoxylin staining. Negative controls have been performed by removing the primary antibody [21]. To verify nonspecific background immunostaining, slices were incubated with either primary or secondary Ab alone. No staining was observed in these controls, which proved that the immunoreactions were positive in all experiments performed. Immunohistochemical images were acquired by using light microscopy (Leica Microsystem) connected to a high-resolution digital camera DFC425B Leica (Leica Microsystem).

### 2.6. Immunofluorescence Analysis

The hDPSCs alone or hDPSCs cultured on DDP of all experimental groups were processed for immunofluorescence experiments. Primary monoclonal antibodies anti-human ALP (1:200, rabbit) (Santa Cruz Biotechnology, Dallas, TX, USA), anti-human RUNX2 (1:200, rabbit) (Santa Cruz Biotechnology), anti-human COL1A1 (1:100, rabbit) (Santa Cruz Biotechnology), anti-human Vinculin (1:200, rabbit) (Santa Cruz Biotechnology), anti-human DMP1 (1:200, rabbit) (Santa Cruz Biotechnology) and anti-human DSPP (1:200, rabbit) (Santa Cruz Biotechnology) were used followed by incubation with Alexa Fluor 568 conjugated goat anti rabbit secondary antibodies (1:200; ThermoFisher, Life Technologies, Monza, Italy) for 1 h at 37 °C. Subsequently, cells were incubated with AlexaFluor 488 phalloidin green fluorescence conjugate (1:200; ThermoFisher, Life Technologies) to evidence cytoskeleton actin. Cell nuclei were stained with TOPRO (1:200; ThermoFisher, Life Technologies) for 1 h at 37 °C. Glass coverslips were placed face down on glass slides and mounted with Prolong antifade (ThermoFisher, Life Technologies). Samples were observed by means of a Zeiss LSM800 confocal system connected to an inverted Zeiss Axiovert 200 microscope equipped with a Plan Neofluar oil-immersion objective.

### 2.7. Western Blot Analysis

Proteins extracted from all experimental groups were processed as previously described [22]. Briefly, 30 µg of proteins were resolved on SDS–PAGE gel and subsequently transferred to nitrocellulose sheets using a semidry blotting apparatus. Sheets were saturated for 120 min at room temperature in blocking buffer (1xTBS, 5% milk, 0.1% Tween-20) and incubated overnight at 4 °C in blocking buffer containing primary antibodies ALP (1:500, rabbit) (Santa Cruz Biotechnology), RUNX2 (1:500, rabbit) (Santa Cruz Biotechnology), COL1A1 (1:500, rabbit) (Santa Cruz Biotechnology), Vinculin (1:500, rabbit) (Santa Cruz Biotechnology), DMP1 (1:500, rabbit) (Santa Cruz Biotechnology), and DSPP (1:50, rabbit) (Santa Cruz Biotechnology). β-Actin (1:750, mouse) (Santa Cruz Biotechnology) was used as a loading control. After four washes in TBS containing 0.1% Tween-20, samples were incubated for 60 min at room temperature with peroxidase-conjugated secondary antibody diluted as 1:1000 in 1 × TBS that contained 2.5% milk and 0.1% Tween-20. Bands were visualized and quantified by the ECL method with Alliance 2.7 (UVItec Limited, Cambridge, UK).

### 2.8. Statistical Analysis

All experiments were performed in triplicate. All data are expressed as mean ± standard deviation. Statistical analyses were performed using the Student’s unpaired *t*-test for comparisons of two groups and one-way ANOVA followed by the Bonferroni method for comparisons of three or more groups. Values of *p* < 0.05 were considered statistically significant.

## 3. Results

### 3.1. Characterization of DDP and DP

**Figure 1:**
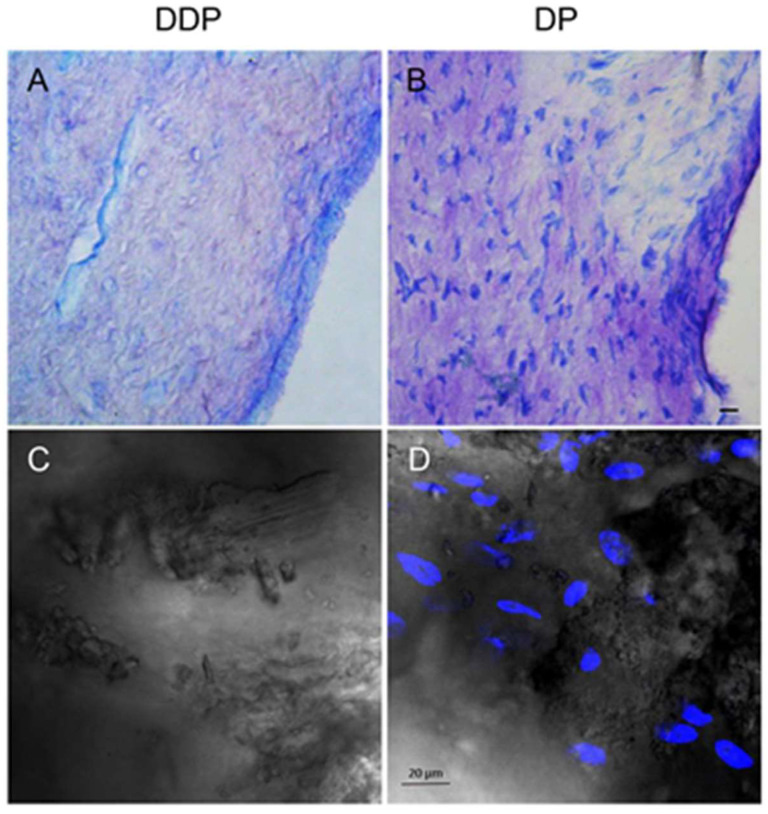
Morphological and cell distribution analyses in DDP and DP. (**A**,**B**) Images of toluidine blue staining of (**A**) DDP and (**B**) DP. Cell nuclei are clearly visible in DP sample when compared to DDP. Representative images are shown. (**C**,**D**) Images obtained at confocal laser scanning microscopy showed the presence of nuclei stained in blue in the DP sample, while in DDP the staining for cell nuclei was negative. Scale bar = 20 μm.

The fresh dental pulp was extracted from human wisdom teeth as previously described. The histologic toluidine blue analysis (Figure 1A,B) revealed that the cellular components of the dental pulp were completely removed after the decellularization process. The absence of nuclear contents was demonstrated by immunofluorescence staining against TOPRO (Figure 1C,D).

### 3.2. Morphological Analysis of Recellularized DDP

**Figure 2:**
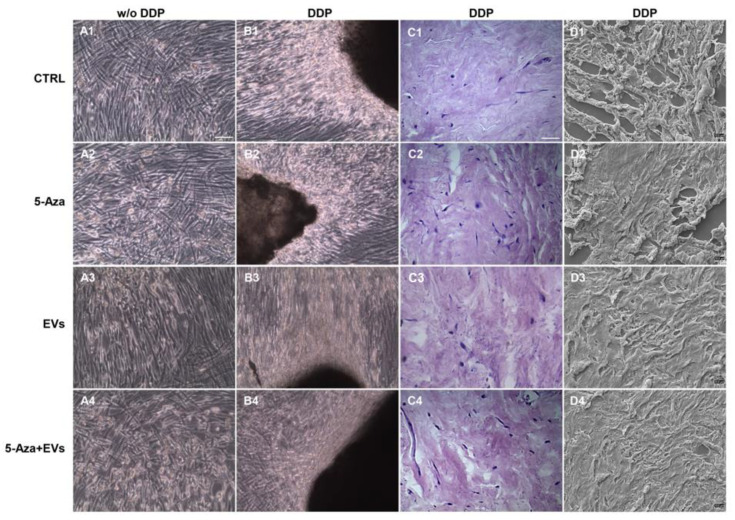
Morphological evaluation of hDPSCs cultured with and without DDP. (**A1**–**A4**) Human DPSCs cultured without DDP observed under light microscopy in all conditions considered in the study. (**A1**) Cells cultured with basal medium (CTRL), (**A2**) cells cultured with 5-Aza (5-Aza), (**A3**) cells cultured with EVs (EVs), and (**A4**) cells cultured with EVs+5-Aza (EVs+5-Aza). (**B1**–**B4**) Human DPSCs cultured on DDP observed under light microscopy in all conditions considered in the study. (**B1**) Cells cultured on DDP with basal medium (DDP-CTRL), (**B2**) cells cultured on DDP with 5-Aza (DDP-5-Aza), (**B3**) cells cultured on DDP and treated with EVs (DDP-EVs), and (**B4**) cells cultured on DDP with EVs+5-Aza (DDP-EVs+5-Aza). (**C1**–**C4**) Haematoxylin-eosin staining of hDPSCs cultured on DDP in all conditions considered in the study. (**C1**) Cells cultured on DDP with basal medium (DDP-CTRL), (**C2**) cells cultured on DDP with 5-Aza (DDP-5-Aza), and (**C3**) cells cultured on DDP and treated with EVs (DDP-EVs), (**C4**) Cells cultured on DDP with EVs+5-Aza (DDP-EVs+5-Aza). (**D1**–**D4**) Human DPSCs cultured on DDP observed via scanning electron microscopy in all conditions considered in the study. (**D1**) Cells cultured on DDP with basal medium (DDP-CTRL), (**D2**) cells cultured on DDP with 5-Aza (DDP-5-Aza), (**D3**) cells cultured on DDP and treated with EVs (DDP-EVs), and (**D4**) cells cultured on DDP with EVs+5-Aza (DDP-EVs+5-Aza). Scale bar: 100 µm.

Cells were cultured under different culture conditions: with basal medium (CTRL), 5-Aza, EVs, and EVs+5-Aza in the presence or absence of DDP. Morphological analyses performed under light microscopy showed a fibroblast-like morphology growth in the monolayer for cells cultured without DDP for all considered conditions (Figure 2A1–A4). Cells cultured with DDP showed a high cellular density (Figure 2B1–B4). To demonstrate DDP recellularization, a histologic analysis was performed on DDP cultures with hDPSCs (Figure 2C1–C4). In particular, hDPSCs were visible at the periphery of DDP in samples maintained with 5-Aza+EVs, when compared to samples treated only with EVs or 5-Aza. SEM analyses were used to confirm the recellularization process of DDP by hDPSCs (Figure 2D1–D4).

### 3.3. Expression of ALP, RUNX2, COL1A1, Vinculin, DMP1, and DSPP

**Figure 3:**
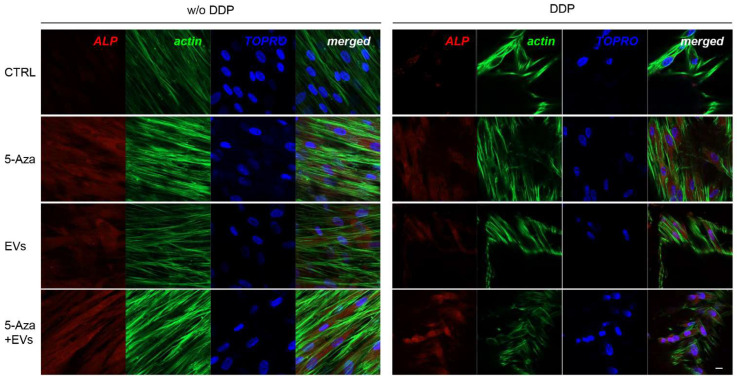
Immunofluorescence analysis of ALP expression. Left panel shows the images of hDPSCs (CTRL), hDPSCs+5-Aza (5-Aza), hDPSCs+EVs (EVs), and hDPSCs+5-Aza+EVs (5-Aza+EVs) cultured without DDP. Right panel shows the images of hDPSCs (CTRL), hDPSCs+5-Aza (5-Aza), hDPSCs+EVs (EVs), and hDPSCs+5-Aza+EVs (5-Aza+EVs) cultured without DDP. Red fluorescence: ALP marker; green fluorescence: cytoskeleton actin; blue fluorescence: cell nuclei using TOPRO; merged image: overlap of all above mentioned channels. Representative images are shown. Scale bar: 20 µm.

**Figure 4:**
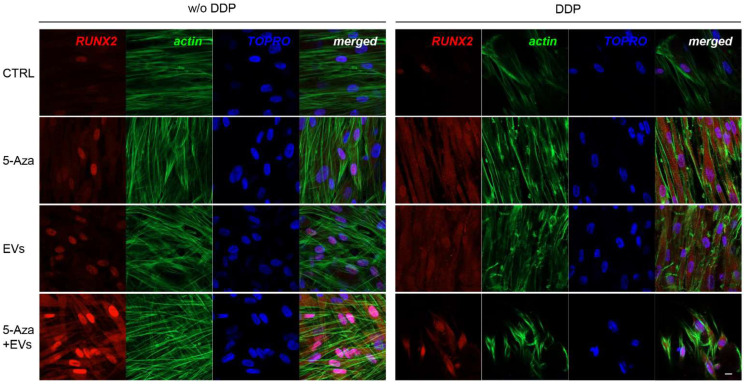
Immunofluorescence analysis of RUNX2 expression. Left panel shows the images of hDPSCs (CTRL), hDPSCs+5-Aza (5-Aza), hDPSCs+EVs (EVs), and hDPSCs+5-Aza+EVs (5-Aza+EVs) cultured without DDP. Right panel shows the images of hDPSCs (CTRL), hDPSCs+5-Aza (5-Aza), hDPSCs+EVs (EVs), and hDPSCs+5-Aza+EVs (5-Aza+EVs) cultured without DDP. Red fluorescence: ALP marker; green fluorescence: cytoskeleton actin; blue fluorescence: cell nuclei using TOPRO; merged image: overlap of all above mentioned channels. Representative images are shown. Scale bar: 20 µm.

**Figure 5:**
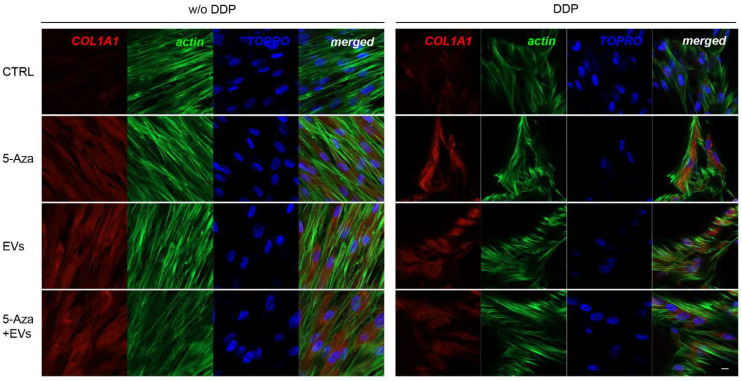
Immunofluorescence analysis of COL1A1 expression. Left panel showes the images of hDPSCs (CTRL), hDPSCs+5-Aza (5-Aza), hDPSCs+EVs (EVs), and hDPSCs+5-Aza+EVs (5-Aza+EVs) cultured without DDP. Right panel shows the images of hDPSCs (CTRL), hDPSCs+5-Aza (5-Aza), hDPSCs+EVs (EVs), and hDPSCs+5-Aza+EVs (5-Aza+EVs) cultured without DDP. Red fluorescence: ALP marker; green fluorescence: cytoskeleton actin; blue fluorescence: cell nuclei using TOPRO; merged image: overlap of all above mentioned channels. Representative images are shown. Scale bar: 20 µm.

**Figure 6:**
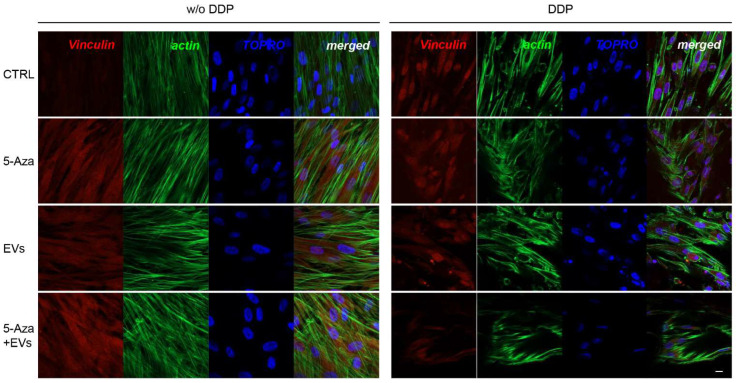
Immunofluorescence analysis of Vinculin expression. Left panel shows the images of hDPSCs (CTRL), hDPSCs+5-Aza (5-Aza), hDPSCs+EVs (EVs), and hDPSCs+5-Aza+EVs (5-Aza+EVs) cultured without DDP. Right panel shows the images of hDPSCs (CTRL), hDPSCs+5-Aza (5-Aza), hDPSCs+EVs (EVs), and hDPSCs+5-Aza+EVs (5-Aza+EVs) cultured without DDP. Red fluorescence: ALP marker; green fluorescence: cytoskeleton actin; blue fluorescence: cell nuclei using TOPRO; merged image: overlap of all above mentioned channels. Representative images are shown. Scale bar: 20 µm.

**Figure 7:**
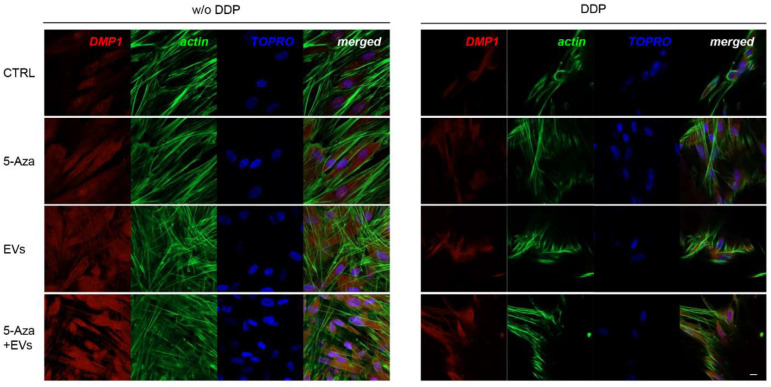
Immunofluorescence analysis of DMP1 expression. Left panel shows the images of hDPSCs (CTRL), hDPSCs+5-Aza (5-Aza), hDPSCs+EVs (EVs), and hDPSCs+5-Aza+EVs (5-Aza+EVs) cultured without DDP. Right panel shows the images of hDPSCs (CTRL), hDPSCs+5-Aza (5-Aza), hDPSCs+EVs (EVs), and hDPSCs+5-Aza+EVs (5-Aza+EVs) cultured without DDP. Red fluorescence: ALP marker; green fluorescence: cytoskeleton actin; blue fluorescence: cell nuclei using TOPRO; merged image: overlap of all above mentioned channels. Representative images are shown. Scale bar: 20 µm.

**Figure 8:**
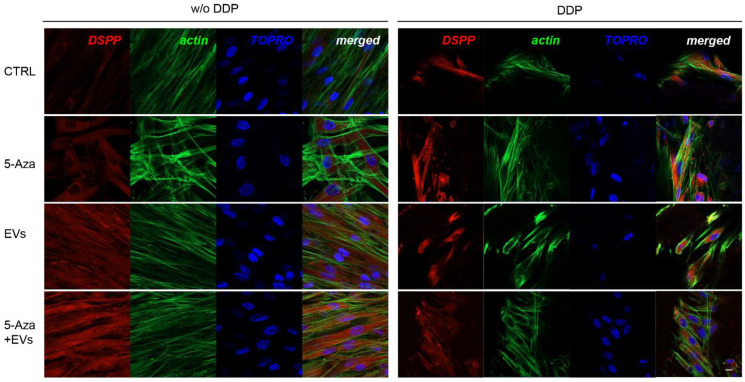
Immunofluorescence analysis of DSPP expression. Left panel shows the images of hDPSCs (CTRL), hDPSCs+5-Aza (5-Aza), hDPSCs+EVs (EVs), and hDPSCs+5-Aza+EVs (5-Aza+EVs) cultured without DDP. Right panel shows the images of hDPSCs (CTRL), hDPSCs+5-Aza (5-Aza), hDPSCs+EVs (EVs), and hDPSCs+5-Aza+EVs (5-Aza+EVs) cultured without DDP. Red fluorescence: ALP marker; green fluorescence: cytoskeleton actin; blue fluorescence: cell nuclei using TOPRO; merged image: overlap of all above mentioned channels. Representative images are shown. Scale bar: 20 µm.

ALP, RUNX2, COL1A1, Vinculin, DMP1, and DSPP positive cells were detected by means of immunofluorescence analysis using confocal laser scanning microscopy. However, the positivity varied for intensity and distribution patterns depending on the target proteins. Human DPSCs cultured without DDP in basal medium for ALP, RUNX2, COL1A1, Vinculin, DMP1, and DSPP showed a negative expression (Figure 3, Figure 4, Figure 5, Figure 6, Figure 7 and Figure 8), while the treatment with EVs or 5-Aza showed an increased positivity of red fluorescence at the cytoplasmic level; only RUNX2 showed a nuclear localization (Figure 4). The co-treatment with EVs and 5-Aza showed a marked positivity for all markers in all considered conditions (Figure 3, Figure 4, Figure 5, Figure 6, Figure 7 and Figure 8).

Human DPSCs cultured on DDP with basal medium (DDP-CTRL) showed a negative expression of ALP (Figure 3) and a weak positive reaction was observed for RUNX2, COL1A1, Vinculin, DMP1, and DSPP (Figure 4, Figure 5, Figure 6, Figure 7 and Figure 8).

The positive expression found in hDPSCS co-treated with EVs and 5-Aza was less than samples treated with EVs or 5-Aza alone (Figure 3, Figure 4, Figure 5, Figure 6, Figure 7 and Figure 8).

### 3.4. Protein Expression of Dentinogenesis Tissue Markers in hDPSCs Cultured with and without of DDP and Odontoblast-Like Cellular Morphology

**Figure 9:**
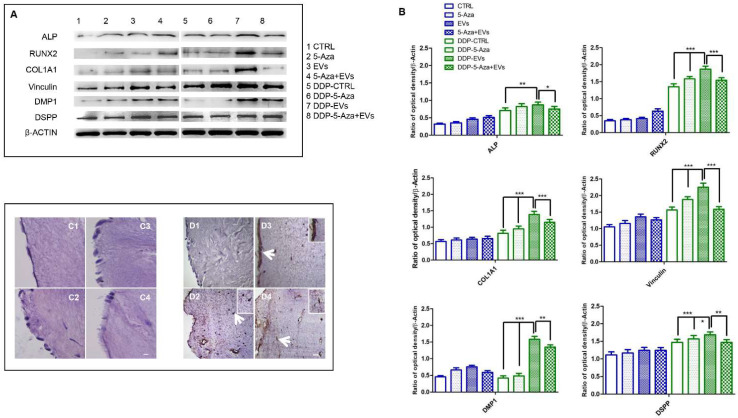
Odontoblast differentiation. (**A**) Specific Western blot bands of ALP, RUNX2, COL1A1, Vinculin, DMP1. and DSPP evaluated in all sample groups. (**B**) Densitometric analyses of ALP, RUNX2, COL1A1, Vinculin, DMP1, and DSPP. β-actin was used as loading control. Haematoxylin-eosin staining in all sample groups. Haematoxylin-eosin staining of (**C1**) hDPSCs cultured on DDP, (**C2**) hDPSCs cultured on DDP with 5-Aza, (**C3**) hDPSCs with EVs, and (**C4**) hDPSCs cultured on DDP with EVs+5-Aza. Immunoistochemical analysis of DSPP expression in (**D1**) hDPSCs cultured on DDP, (**D2**) hDPSCs cultured on DDP with 5-Aza, (**D3**) hDPSCs with EVs, and (**D4**) hDPSCs cultured on DDP with EVs+5-Aza. Arrows indicate the magnified inset area. Scale bar: 100 µm. Significant statistical differences: ALP DDP-EVs vs. DDP-CTRL ** *p* < 0.01, DDP-EVs vs. DDP-5-Aza+EVs * *p* < 0.05; RUNX2, COL1A1, Vinculin, DDP-EVs vs. DDP-CTRL *** *p* < 0.001, DDP-EVs vs. DDP-5-Aza *** *p* < 0.001, DDP-EVs vs. DDP-5-Aza+EVs *** *p* < 0.001; DMP1 DDP-EVs vs. DDP-CTRL *** *p* < 0.001, DDP-EVs vs. DDP-5-Aza *** *p* < 0.001, DDP-EVs vs. DDP-5-Aza+EVs ** *p* < 0.01; DSPP DDP-EVs vs. DDP-CTRL *** *p* < 0.001, DDP-EVs vs. DDP-5-Aza * *p* < 0.05, DDP-EVs vs. DDP-5-Aza+EVs ** *p* < 0.01.

ALP, RUNX2, COL1A1, Vinculin, DMP1, and DSPP were investigated by Western blot analysis. Expression of these proteins was significantly increased in hDPSCs cultured on DDP and treated with EVs. Protein expression showed a significantly high level of RUNX2, COL1A1, and Vinculin in DDP-EVs group when compared to the DDP-5-Aza and DDP-5-Aza+EVs groups. The same results have been obtained in the evaluation of DMP1 and DSPP protein expression, and a statistically significant difference appeared in the DDP-EVs sample compared to the DDP-CTRL and DDP-5-Aza samples (Figure 9A,B). The haematoxilyn-eosin staining showed cells with an odontoblast-like morphology in DDP samples treated with EVs and 5-Aza (Figure 9C1–C4). DSPP expression was performed by immunohistochemistry analysis. Samples showed a positive expression of DSPP in samples treated with 5-Aza and EVs, and co-treated with 5Aza+EVs when compared to the CTRL group. In particular the treatment of EVs alone showed a marked positivity of DSPP when compared to the other samples (Figure 9D1–D4).

## 4. Discussion

The decellularized matrix (DCM) has gained more popularity as a new biomaterial for tissue regeneration. The DCM can be considered as a natural scaffold able to develop a 3D environment with a key role in cell adhesion, migration, and differentiation, and in the regulation of tissue regeneration and homeostasis [23]. Dental pulp regeneration is a primary challenge in the dentistry field, as it plays a key role in maintaining the dental vitality and tooth biomechanical function. Bioengineers have tried to develop novel systems to repair the whole tooth using the transplantation of DPSCs and Stem cells from Human Exfoliated Deciduous Teeth (SHED) with traditional scaffolds, such as hydroxyapatite/tricalcium phosphate, in order to overcome the difficulties during the regeneration of the dentin–pulp-like complex [24,25]. The maintenance of the histological structure of dental pulp is the main outcome of modern dentistry and the use of a DCM derived from dental pulp is able to preserve the physiological pattern of core teeth. ECM represents a noncellular component of the tissue and it can be considered as a direct modulator in regulating many cellular functions, such as adhesion, migration, proliferation, and differentiation. Moreover, it is able to provide the structural support for tissue development as well as regeneration [26]. 

The aim of this study was to develop the DDP scaffold enriched with EVs and 5-Aza for the regeneration of dental pulp and to evaluate the capabilities in the potentiating odontoblastic differentiation of hDPSCs. Human dental pulps (DP)were used in this study as a DCM for the development of the final scaffold with EVs and 5-aza treatment in order to evaluate the in vitro response of hDPSCs. The appropriate decellularization method is necessary to obtain a DCM scaffold [27]. The decellularization process should remove cells and nuclear materials from the tissue and maintain the native histological 3D structure [28]. The decellularization process provides an acellular human DDP scaffold that showed the native dental pulp structure and a reduction in the native DNA content as demonstrated by light microscopy images and immunofluorescence evaluation. The cell homing process may be considered a novel strategy to recruit endogenous cells stimulated by this innovative, biocompatible scaffold that could trigger dental pulp regeneration.

To recellularize the DDP scaffold, hDPSCs were used because of their excellent role in tissue regeneration. hDPSCs showed unique stemness properties, including rapid proliferation, self-renewal ability, and multilineage differentiation potential [29].

During tooth embryological development, the epithelial–mesenchymal interaction induced the hDPSCs to differentiate into odontoblasts to form the primary dentin tissue [30,31]. DPSCs were selected as a cell source for DDP recellularization, as these cells are known to be an excellent candidate for dental pulp regeneration. After recellularization, process hDPSCs are able to attach, proliferate, and migrate on the DDP scaffold, as these cells have demonstrated their suitability for cell binding and growth. The light microscopy and SEM images demonstrated the capacity of hDPSCs to recolonize the DDP scaffold.

Previous studies have already examined the role of hDPSCs in the dental pulp tissue regeneration in tooth root fragments through a scaffold-free strategy using SHED-derived cells. The major difficulty was developing the complex histological dental pulp structure that possesses highly organized physiologic patterns [32].

EVs derived from MSCs have been studied for their beneficial effects on tissue regeneration due to their paracrine action [33,34]. Several molecular factors are released by EVs which promote cell recruitment with a significant potential role for endogenous tissue repair and regeneration [35,36]. Our previous research also showed that EVs can enhance tissue regeneration when combined with a 3D scaffold and represent a cell-free therapy approach [37]. Moreover, EVs derived from hDPSCs could promote cellular functions and thus offer an alternative therapy of a regenerative endodontic approach [38]. In regenerative endodontic therapy, EVs are reported as an ideal biomimetic tool exhibiting potential angiogenesis, which induces stem-cell recruitment into the root canal followed by differentiation [39,40]. Exosomes, by carrying cell-type specific, biological molecules, such as proteins, mRNA, and microRNA, are fundamental for intercellular communications during tissue formation and repair [41]. In particular, microRNAs play important roles in stem-cell differentiation and microRNAs promote the odontogenic differentiation via the TGFβ1/smads signaling pathway by downregulating the inhibitory molecule LTBP1 in hDPSCs [40,42]. It is a cell-free approach and an alternative to cell transplantation strategies, avoiding the use of whole cells for implantation and their concerns.

To promote the odontogenic cell differentiation, 5-Aza was used in our experimental approach. Deqian Zhang et al. reported that 5-Aza suppressed the cell proliferation ability and enhanced the odontogenic differentiation potential of hDPSCs [9].

To explore the effect of EVs and 5-Aza on the odontogenic differentiation potential of hDPSCs seeded on DDP scaffold, we examined the expression of ALP, RUNX2, COL1A1, Vinculin, DMP1, and DSPP by means Western blot analysis. The DDP matrix enriched with EVs showed a positive expression of all considered markers when compared to the DDP enriched with 5-Aza alone or 5-Aza+EVs. ALP is considered as a critical factor during the early stages of bone and dental tissue formation.

RUNX2 is a transcription factor that plays a key role in osteoblast and odontoblast differentiation and is able to modulate the development and maintenance of bone and teeth [43,44].

Type I collagen is the major protein in the extracellular dentin matrix that regulates the expression of DMP1, expressed mainly in the active odontoblasts [45].

Vinculin is a protein implicated in a transmembrane mechanism that controls odontoblast differentiation [46,47]. DSPP and DMP1 are considered as odontogenic markers that promote the first step of the dentin matrix collagen mineralization process [48].

It is well known that 5-Aza had an inhibitory effect on hDPCs and suppressed the cell growth rate from the third day onward. Previous studies reported that 5-Aza could upregulate the expression levels of DSPP, DMP1, RUNX2, DLX5, and OSX, promote ALP activity, and accelerate the formation of mineralized nodules [9]. 5-Aza treatment is able to induce the odontogenic differentiation f hDPSCs without the use of odontogenic medium, reduces the DNA methylation levels of some odontogenic differentiation-associated genes, such as ALP or DLX5, and upregulates their expression in hDPSCs.

Our results showed that EVs and 5-Aza enrichment upregulated the expression levels of ALP, RUNX2, COL1A1, Vinculin, DMP1, and DSPP in hDPSCs cultured on DDP as demonstrated by the protein expression and immunofluorescence analyses.

In the present study, EVs and 5-Aza treatment on hDPSCs seeded on DDP induced a high expression of odontogenic and osteogenic markers compared to the hDPSCs without the decellularized scaffold. The findings of this study suggested that DDP has the potential role to be a promising, tool-promoting strategy for dental pulp regeneration. Although recent studies reported the development of hydrogels composed by DDP of bovine and human origin [49,50], the present study demonstrates the benefit of using DDP in its intact form for dental pulp regeneration via the use of mesenchymal stem cells and extracellular vesicles. The use of a stem-cell approach for dental pulp regeneration has shown immense potential for rebuilding the complex histological structure of the native pulp with a highly organized pattern [51].

More studies are necessary to further elucidate the role of the DDP scaffold in cellular recruitment and in the regulation of the odontogenic differentiation process of hDPSCs. Our findings suggest that DDP enriched with hDPSCs and EVs showed a high potentiality to provide a promising scaffold in dental pulp regeneration, promoting hDPSC odontogenic differentiation.

The use of decellularized scaffolds to design a novel biomaterial overcome some difficulties in the endodontic regenerative practice as the DDP may directly fill the root canal of the teeth and have an optimized architecture to allow endogenous cell colonization and proliferation into the intracanal space.

## 5. Conclusions

In conclusion, this study showed that EV treatment could improve the odontogenic differentiation capacity of hDPSCs by increasing the expression of odontogenic markers and transcription factors, new avenues for research in dental pulp repair and regeneration open. This novel approach might hold great translational potential in dental pulp regeneration, which could be an alternative clinical therapeutic strategy for traditional endodontic treatment.

## Data Availability

Data are available upon request to the corresponding authors.

## Abbreviations

DDP	Decellularized Dental Pulp
5-Aza	5-Aza-2′-deoxycytidine
EVs	Extracellular Vesicles
hDPSCs	human Dental Pulp Stem Cells
dECMs	decellularized Extracellular Matrices
DCM	Decellularized matrix
DNMTs	DNA methyltransferases
MSCs	Mesenchymal Stem Cells
SHED	Human Exfoliated Deciduous Teeth
SEM	Scanning Electron Microscopy
COL1A1	Collagen 1A1
DSPP	Dentin Sialophosphoprotein
DMP1	Dentin Matrix Acidic Phosphoprotein 1
RUNX2	Runt-Related Transcription Factor 2
OSX	Osterix
ALP	Alkaline Phosphatase
MSCGM-CD	Mesenchymal Stem Cell Growth Medium-Chemically Defined
DP	Dental pulps
DPBS	Dulbecco’s Phosphate-Buffered Saline
PFA	Paraformaldehyde
